# Optimization of the Ex Situ Biomethanation of Hydrogen and Carbon Dioxide in a Novel Meandering Plug Flow Reactor: Start-Up Phase and Flexible Operation

**DOI:** 10.3390/bioengineering11020165

**Published:** 2024-02-08

**Authors:** Kevin Hoffstadt, Marcell Nikolausz, Simone Krafft, Maria Letícia Bonatelli, Vivekanantha Kumar, Hauke Harms, Isabel Kuperjans

**Affiliations:** 1 Institute NOWUM-Energy, University of Applied Sciences Aachen, Heinrich-Mussmann-Str. 1, 52428 Juelich, Germany; vivekanantha.kumar@alumni.fh-aachen.de (V.K.); kuperjans@fh-aachen.de (I.K.); 2 Department of Microbial Biotechnology, Helmholtz Centre for Environmental Research–UFZ, Permoserstr. 15, 04318 Leipzig, Germany; marcell.nikolausz@ufz.de (M.N.); maria.bonatelli@ufz.de (M.L.B.); 3 Department of Applied Microbial Ecology, Helmholtz Centre for Environmental Research–UFZ, Permoserstr. 15, 04318 Leipzig, Germany; hauke.harms@ufz.de

**Keywords:** methanation, plug flow reactor, bubble column, biomethane, power-to-gas, P2G

## Abstract

With the increasing use of renewable energy resources for the power grid, the need for long-term storage technologies, such as power-to-gas systems, is growing. Biomethanation provides the opportunity to store energy in the form of the natural gas-equivalent biomethane. This study investigates a novel plug flow reactor that employs a helical static mixer for the biological methanation of hydrogen and carbon dioxide. In tests, the reactor achieved an average methane production rate of 2.5 $\frac{L_{{CH}_{4}}}{L_{R}*d}$ (methane production [L_CH4_] per liter of reactor volume [L_R_] per day [d]) with a maximum methane content of 94%. It demonstrated good flexibilization properties, as repeated 12 h downtimes did not negatively impact the process. The genera *Methanothermobacter* and *Methanobacterium* were predominant during the initial phase, along with volatile organic acid-producing, hydrogenotrophic, and proteolytic bacteria. The average ratio of volatile organic acid to total inorganic carbon increased to 0.52 ± 0.04, while the pH remained stable at an average of pH 8.1 ± 0.25 from day 32 to 98, spanning stable and flexible operation modes. This study contributes to the development of efficient flexible biological methanation systems for sustainable energy storage and management.

## 1. Introduction

Predictions of the dramatic consequences of the climate crisis demand rapid action. There is an urgent need to drastically reduce the emission of carbon dioxide, which in turn requires shifting to renewable energy sources such as wind and solar power [1,2]. However, as these intermittent resources grow in importance, power generation will become more dependent on changes in the weather, leading to grid instability [3]. This challenge becomes even greater with the rise of electric vehicles and electric heating systems, which put additional stress on power grids [4,5].

Energy storage systems, such as batteries and mechanical energy storage technologies, can help to stabilize grids by storing renewable energy. Decentralized storage in homes and businesses reduces our reliance on fossil fuels [6,7]. Centralized systems increase grid flexibility, countering periods of low renewable energy availability [8].

There are short-term and long-term storage options. One promising long-term approach is based on the power-to-gas (P2G) method, which uses excess electricity to produce hydrogen or, after further conversion, methane, which can be integrated into existing natural gas networks or hydrogen gas networks [9,10].

The process of chemical methanation uses catalysts of the Sabatier process. Alternatively, a biotechnological route uses methanogenic archaea, microorganisms capable of producing methane. Biomethanation can occur directly, through hydrogenotrophic methanogens converting hydrogen and carbon dioxide, or indirectly, through homoacetogens producing acetate, which is later converted into methane and carbon dioxide by acetoclastic methanogens [11].

The reactor types used for biomethanation, such as continuous stirred-tank reactors and trickle-bed reactors, present different advantages and challenges, though all rely on efficient hydrogen mass transfer between reaction phases [12,13]. In general, continuous stirred-tank reactors achieve a higher methane production rate (MPR) with high parasitic energy, while trickle-bed reactors achieve a lower, but still acceptable, MPR with lower levels of parasitic energy. A novel biofilm plug flow reactor has also demonstrated favorable properties in this regard, though scaling up may pose a challenge [14].

Another challenge is ensuring that biological methanation can be applied flexibly. It has been shown that certain methanogenic microorganisms can survive long periods of starvation, which makes them suitable for flexible operation [15,16]. While flexible biomethanation has already been tested in trickle-bed reactors and continuous stirred-tank reactors, these reactor types are often not optimized for such operations [17,18,19]. Therefore, the development of a new kind of reactor is desirable, as outlined in our previous work. This novel design is tested in the study outlined in this paper [20].

In the study described below, we inoculated a plug flow reactor and operated it at 55 °C to establish a stable methanation process. Its microbial structure was investigated during the start-up phase. In addition, we tested the effects of intermittent feeding on the stability of the process, pausing and continuing the supply of feed gas to mimic flexible operation with renewable energy sources.

## 2. Materials and Methods

### 2.1. Reactor Setup

**Figure 1:**
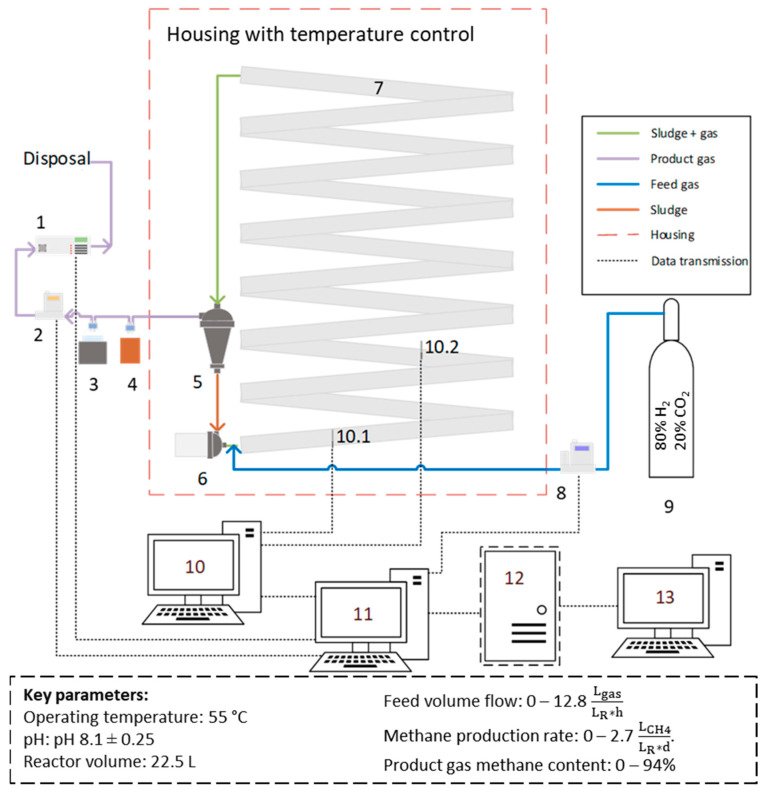
Schematic diagram of the reactor. 1: gas analyzer (Sensors: CH_4_, CO_2_, H_2_, O_2_, SSM 6000, Pronova Analysentechnik GmbH & Co. KG, Berlin, Germany); 2: gas flow meter (F-101EI-AGD-00-K, Bronkhorst Deutschland Nord GmbH, Kamen, Germany); 3: chiller at 5 °C (WineCase One, CASO, Arnsberg, Germany), 1 L borosilicate bottle (VWR International GmbH, Darmstadt, Germany); 4: 0.5 L borosilicate bottle as sludge container (VWR International GmbH, Darmstadt, Germany); 5: gas–liquid separator (Hydrocyclone Filter 1″, Alfaturbo, Plastica Alfa, Caltagirone, Italy); 6: pump (LS543238, Lilie GmbH & Co. KG, Besigheim, Germany); 7: plug flow reactor with integrated helical structure; 8: gas flow controller (F-201CI-5K0-AGD-00-V, Bronkhorst Deutschland Nord GmbH, Kamen, Germany); 9: gas bottle (80% H_2_, 20% CO_2_, Westfalen AG, Muenster, Germany); 10: Raspberry Pi 4 Model B for temperature measurement; 10.1 temperature sensor (ds18b20); 10.2 temperature sensor (ds18b20); 11: Raspberry Pi 4 Model B for data processing; 12: data server; 13: computer for monitoring.

The plug flow reactor design includes a 50 mm diameter polyvinyl chloride tube system with an integrated static mixer in the form of a helical structure to enhance its properties. The length of the tubes with the static mixers is 14 m. The design also incorporates gas analysis devices and a data processing unit for comprehensive data interpretation. A diagram depicting the reactor system can be found in Figure 1.

A more detailed description of the design and construction process can be found in our previous article [20]. The gas analyzer and the gas flow controller used in this previous study were replaced by an SSM 6000 (Pronova Analysentechnik GmbH & Co. KG, Berlin, Germany) and an F-201CI (Bronkhorst Deutschland Nord GmbH, Kamen, Germany), respectively. The liquid phase was recirculated at 4.3 L/min. The process was conducted at 55 ± 1 °C, which is the operating temperature of the biogas plant of the inoculum.

### 2.2. Inoculum

The active anaerobic sludge used as an inoculum for the reactor was obtained from an industrial-scale thermophilic biogas plant (55 °C) located in North Rhine-Westphalia, Germany (coordinates: 51.881640, 6.578983) that was fed with cow and horse manure. Due to the high viscosity of the sludge and its high fiber content, 2 kg of sludge was diluted with 1 kg of water and sieved through a colander. This process was repeated 16 times and the filtrate was collected each time. While sieving was sufficient to eliminate most of the fiber content, sand was still present. To protect the recirculation pump of the reactor, the sand content had to be reduced. Therefore, the filtrate was centrifuged for 2 min at 4 °C (900 mL in 1 L bottles, 4256× *g*, Rotor S4×-Universal Eppendorf centrifuge 5910 Ri, Eppendorf, Hamburg, Germany). The resulting pellet containing a high sand content was discarded, and the supernatant was collected and centrifuged again for 30 min at the same speed. The supernatant of the second centrifugation step was disposed of and the pellet was dissolved in minimal medium equal to the mass of the discarded supernatant. The solution after dissolving the pellet had a pH of 7.8, a volatile organic acid (VOA) to total inorganic carbonate (TIC) ratio of 0.174, and an OD of 10.2.

### 2.3. Minimal Medium

A minimal medium was used for inoculum preparation and as a broth used in the reactor. It has been reported that ammonia can be limiting in biomethanation and is optimal at a concentration higher than 1 g/L but below 1.5 g/L [21,22]. Therefore, the N concentration was set at 1 g/L and supplied as urea, which can be hydrolyzed to ammonia by urease, with diammonium phosphate as an additional ammonium source. Diammonium phosphate served also as the sole phosphate source.

The optimal C:N:P:S ratio for the biogas process is documented as 600:15:5:1 [23]. Since the sludge was from a biogas plant, this ratio was applied to the methanation process. The actual C ratio was unknown and, in our case, mainly comes from CO_2_. Therefore, phosphorus was added to the medium based on the ammonia concentration, at a concentration of 0.3 g/L. Sulfur was added in the form of sodium sulfide, as sulfide can be used by archaea [24]. The sulfide and the trace element solutions were prepared according to previous studies [25]. Then, 1 mL/L of sterile trace element solution SL-10 (from DSMZ medium 320) and selenite-tungstate solution (from DSMZ medium 385) were added. Stock solutions of trace element solution SL-10 and selenite-tungstate solution were stored at room temperature. The stock solution of NaS 9xH_2_O was stored at −20 °C.

The urea diammonium phosphate solution was prepared fresh on each day of supplementation. The pH was set to pH 8 using HCL and NaOH. The detailed recipe and preparation of the media are described in the Supplementary Materials (Text S1, Tables S1–S3). The 1 × medium contained 0.878 g/L urea, 0.4263 g/L diammonium phosphate, and 0.375 g/L NaS 9xH_2_O.

### 2.4. Initial Phase

The reactor was inoculated with 17 L of the prepared inoculum. The cultivation was executed anaerobically under thermophilic conditions at 55 °C. The initial feed volume flow was 5 L/h of a gas mixture consisting of 80% hydrogen and 20% carbon dioxide. This corresponds to a hydrogen loading rate of 4.3$\frac{L_{H_{2}}}{L_{R}*d}$ and a carbon dioxide loading rate of 1.1 $\frac{L_{{CO}_{2}}}{L_{R}*d}$ (5.4$\frac{L_{gas}}{L_{R}*d}$ gas loading rate in total).

The process was not stable at the beginning, as indicated by signs of acidification and a low MPR. Therefore, 4 L of the prepared inoculum, stored at room temperature, was added on day 13 and 8 L on day 28. Between days 28 and 32 the gas feed was paused. The first nutrient supplementation was supplied on day 23, consisting of 50 mL of 50 × concentrated minimal medium. Additional supplements were provided twice per week from day 32 onward. The amount of supplementary solution added to the process was increased and was calculated based on the produced amount of methane. Stoichiometrically, two moles of water are produced for each mole of methane. The amount of produced water was calculated, and supplements were added to counteract dilution by the produced water. The reactor was supplemented using a 50 × minimal medium. An additional 100 mL of 1 × minimal medium was added to flush the inlet valve after supplementation.

After day 32, the process was observed to be stable, and the loading rate was increased stepwise by 1 L/h per day. Between days 48 and 52 the gas feed rate was not increased to avoid a destabilization of the process, since the methane concentration did not rise. At day 53 the final hydrogen loading rate of 10.2 $\frac{L_{H_{2}}}{L_{R}*d}$ and a final carbon dioxide loading rate of 2.6 $\frac{L_{{CO}_{2}}}{L_{R}*d}$ was set. The initial phase was operated until day 73 and was followed by flexible feeding regimes.

### 2.5. Flexible Phase

The aim of the flexible operation was to simulate the use of renewable energy from the German market to produce hydrogen for the reactor and to test the process’s resilience to flexible operation. Improved nutrient supplementation and temperature control were used in accordance with the initial phase of the study. Two flexible feeding regimes were developed. The first of these was based on data for combined photovoltaic (PV), hydropower, biomass, and wind energy obtained from German electricity market data from smard.de [26]. The electric power production data for a single day (31 March 2023) was downloaded and smoothened between 21:30 h and 23:30 h to avoid large steps in the load rate between 23:30 h and 00:00 h the following day. The highest value was set to 100% (12.8 $\frac{L_{gas}}{L_{R}*d}$). Since wind and PV were the most variable power sources, this option is referred to hereafter as the “Wind + PV regime”. The feeding regime can be found in the Supplementary Materials (Table S4).

The second regime was based only on PV data, which also was developed based on data from smard.de [26]. Data from three days (3–5 April 2023) were downloaded and a curve of the average values for each daytime was calculated. The highest value was set as 100% (12.8 $\frac{L_{gas}}{L_{R}*h}$). This regime was called the PV regime. The feeding regime can be found in the Supplementary Materials (Table S5). The operating temperature and recirculation were not changed during the flexible phase.

### 2.6. Analytical Methods

To determine the DM content, the mass of the samples was determined before and after drying at 105 °C. The difference in mass between the fresh and dried samples corresponds to the total solids (TS) value and was measured using a precision scale (ACJ 300-4M, KERN & SOHN GmbH, Balingen-Frommern, Germany).

Total nitrogen content (N total), total phosphorus (P total), and total organic carbon (TOC) were measured, using photometric methods, with a DR2800 photometer (Hach Lange GmbH, Duesseldorf, Germany) and the respective reagents from Hach: LCK238 for N total, LCK350 for P total, and LCK380 for TOC. The manufacturer’s instructions were followed to ensure accurate and reliable results.

The VOA/TIC was measured to detect volatile organic acid formation and buffer capacity. This measurement was conducted using an automated titrator (AT 1000 Series, Hach Lange GmbH, Duesseldorf, Germany). Optical density at 600 nm was measured using a spectrophotometer (Spectronic genesys 10 bio, Thermo Fisher Scientific, Langerwehe, Germany). The sample was diluted with minimal medium and measured to be between A_600_ = 0 and 0.2. Minimal medium was used as a blank. The pH was measured using a Phenomenal^®^ pH 1000 L potentiometer and a Phenomenal^®^ 111 pH electrode (VWR International GmbH, Darmstadt, Germany).

Gas feed flow was controlled using a gas flow controller (F-201CI-5K0-AGD-00-V, Bronkhorst Deutschland Nord GmbH, Kamen, Germany). Gas composition was measured continuously by an SSM 6000 (Pronova Analysentechnik GmbH & Co. KG, Berlin, Germany). Gas product flow was measured using a gas flow meter (F-101EI-AGD-00-K, Bronkhorst Deutschland Nord GmbH, Kamen, Germany). Since the gas meter was calibrated with methane, its values were compensated based on a compensation curve generated using data from the FLUIDAT^®^ database [27]. Details can be found in the Supplementary Materials (Text S2, Figure S1). The MPR was calculated based on the mean values of 1 h intervals using the compensated gas flow, the methane concentration, and the working volume of the reactor.

### 2.7. Microbial Community Analysis

Microbial community analysis was performed on samples taken from the inoculum and during the initial phase of the continuous process. Samples of 1.5 mL were centrifuged at 12,300× *g* for 10 min (Microstar 12, VWR, Darmstadt, Germany). The supernatant was discarded, and the pellets were stored at −20 °C. DNA extraction and one-step amplicon sequencing were executed externally by the company StarSEQ GmbH (Mainz, Germany).

DNA was isolated using a Maxwell^®^ RSC Fecal Microbiome DNA Kit in a Maxwell CSC 48 Instrument (Promega GmbH, Walldorf, Germany). The cells were lysed in a bead mill following the manufacturer’s instructions.

The initial PCR amplification targeted the mcrA gene and the V3–V4 region of the 16S RNA, separately. The following primers consist of Illumina MiSeq-specific overhangs (underlined) and the specific sequence. The mcrA sequence was flanked by mlas (5′-TCGTCGGCAGCGTCAGATGTGTATAAGAGACAG GGTGGTGTMGGDTTCACMCARTA-3′) and mcrA-rev (5′- GTCTCGTGGGCTCGGAGATGTGTATAAGAGACAGCGTTCATBGCGTAGTTVGGRTAGT-3′) [28]. 341f (5′- AATGATACGGCGACCACCGAGATCTACACTCTTTCCCTACACGACGCTCTTCCGATCTCCTACGGGAGGCAGCAGCCTACGGGNGGCWGCAG -3′) and the slightly modified 806Rb Primer 806bR (5′- CAAGCAGAAGACGGCATACGAGATNNNNNNGTGACTGGAGTTCAGACGTGTGCTCTTCCGATCTGACTACNVGGGTWTCTAATCC-3′) were used to flank the V3–V4 region of the 16sRNA [29,30].

Following the initial amplification, the amplicons underwent capillary electrophoresis validation and purification. Subsequently, a second PCR step was carried out to attach Illumina Index1/Index2 adapters for sample indexing. This indexing process remained consistent for both the mcrA and 16S amplicons. The amplicons were pooled equimolarly and purified for downstream analysis.

Sequencing was executed with Illumina MiSeq (paired-end sequencing, read length 300 nt, MiSeq reagent V3, and estimated output of 20–30 M reads). Negative controls of 16S RNA and mcrA primers showed no amplification. Blank control of gDNA isolation confirmed the purity of the isolated DNA. Positive control was executed by StarSEQ GmbH using ZymoBIOMICS^®^ microbial community DNA standard (Zymo Research Corporation). Sequence data processing was conducted in R (version 4.2.0) [31]. DADA2 was used to infer the amplicon sequence variants (ASVs), and phyloseq was used to perform the statistical analysis [32,33]. Samples of the 16S rRNA gene were rarified to an equal depth of 12,247 counts, while samples of the mcrA gene were rarified to 27,385 counts. Demultiplexed raw sequence data were deposited in the EMBL European Nucleotide Archive (ENA) under the study accession number PRJEB70718.

## 3. Results and Discussion

### 3.1. Initial Phase

**Figure 2:**
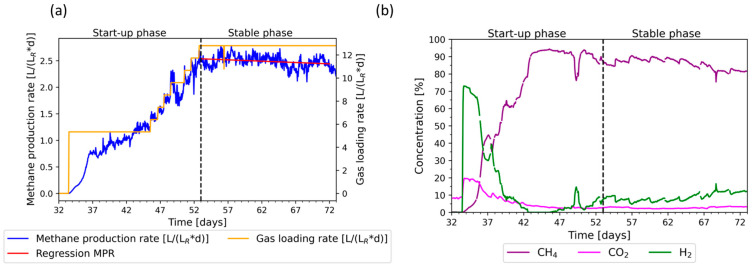
Methane production rate (MPR) during the initial phase after adding inoculum on day 28, feeding with a gas mixture of 20% carbon dioxide and 80% hydrogen. Between days 33 and 45, the gas loading rate of 5.3 $\frac{L}{L_{R}*d}$ was maintained. After day 45 the feed was increased stepwise in increments of 1 L/h per day until day 52, and was kept at 12.8 $\frac{L}{L_{R}*d}$. (**a**) Gas loading rate (orange), methane production rate (blue), and regression line (red); (**b**) product gas concentration of methane (purple), carbon dioxide (magenta), and hydrogen (green).

During the first 31 days, the process was not stable (Supplementary Materials Figure S2). After adding the inoculum again on day 28 and adapting the supplementation of nutrients, the process was stabilized. After day 45 the feed was increased gradually. On day 53 the gas loading rate reached 12.8 $\frac{L_{gas}}{L_{R}*d}$ and was not increased further. The results of the increased loading rate during the initial phase and its impact on the MPR (a) and the gas composition (b) are shown in Figure 2.

The MPR increased with the gas loading rate during the start-up phase until day 53, when a stable process was achieved. The methane concentration also increased until day 45, to a maximum of 95%. In contrast, the hydrogen and carbon dioxide concentrations decreased, while hydrogen was completely consumed between day 43 and day 46. On day 50, the feed was not increased due to a drop in MPR and methane concentration after a short peak. Additionally, the VOA/TIC also dropped on the same day (Figure 5, Section 3.4). This may indicate inhibition due to acidification, which could be caused by a lack of nutrients or by inhibitors added or formed during the process. However, the process recovered, and the feed was increased again starting from day 51. During the stable phase, between days 53 and 72, the average MPR was 2.5 ± 0.1 $\frac{L_{{CH}_{4}}}{L_{R}*d}$ and the methane concentration varied between 81% and 94%, while an increase in the methane concentration after supplementation with nutrients was observed. On the other hand, the hydrogen concentration decreased after supplementation with nutrients, varying between 5% and 17% between day 52 and 73. This indicates the importance of a sufficient supplementation of nutrients.

Trace elements, sulfur, and ammonia are essential for the growth of methanogens. Since sulfur-containing amino acids are essential for the formation of most known amino acid sequences, where the sulfur is also important for proper enzyme folding, sulfur supplementation is inevitable.

Sulfur can be supplemented either by cysteine-HCl, a yeast extract, or by sulfide salts [24,25]. Very few methanogens are able to use sulfate as a sulfur source [24]. In the case of mixed cultures, a previous study has shown that cysteine-HCl might be favorable over sodium sulfide. However, the molar amounts of sulfur in cysteine-HCl and sodium sulfide were not equal in the study [25]. In addition, cysteine HCL is more expensive than sodium sulfide, and it must be considered that amino acids are also a source of carbon. Furthermore, it has been demonstrated that a lack of sufficient sulfur supplementation inhibits the growth and methane production of *Methanobacterium thermoautotrophicum* [34]. Thus, a depletion of sulfide may explain the decreased methane levels in the stable phase.

Certain trace elements are vital for enzyme activity. For instance, nickel is part of the coenzyme F430, which forms the active center of the methyl-coenzyme M reductase enzyme [35]. Thus, supplementing trace elements is necessary for methanation [36,37]. Supplementing trace elements in mixed cultures can be challenging as different organisms may require different elements in varying concentrations [37]. The bioavailability of trace elements may also change over time due to precipitation, dissolution, organic complexation, adsorption, the biotransformation of intermediates containing trace elements, and bio-uptake [38].

Phosphorus and nitrogen are also important nutrients for cell growth. In the literature, ammonia is described as an important nitrogen source for methanogens and below 0.3 g/L inhibition can occur, while the optimum seems to be above 1 g/L [21,39]. In a 2000 L pilot plant the ammonia level was kept at about 0.8 g/L to maintain a stable methanation process. The ammonia not only functions as a nutrient, but also as a buffer.

In our study, N total values between 734 mg/L and 935 mg/L, with an average of 823 mg/L ± 53, and P total values between 85 mg/L and 153 mg/L, with an average of 123 mg/L ± 19, were measured during the whole operation. No trend was detected either for the P total or the N total. Diagrams showing the total nitrogen and phosphate concentrations can be found in the Supplementary Materials (Figure S3).

Phosphorus and nitrogen appear to be adequately supplemented, despite their concentrations being lower than those of the prepared medium. This may be due to the dilution caused by the formation of water or by their fixation in biomass, which settles in areas of the reactor with poor mixing properties. The continuous supplementation of nutrients is favorable and should be applied in future studies. The MPR showed a slightly decreasing trend between days 52 and 73, as shown by the regression line. It could be beneficial to take measurements of ammonia and sulfide to further improve the supplementation of nutrients by identifying possible limitations in the future.

For industrial applications, the MPR is a key factor in determining the size of the plant for biogas or carbon dioxide upgrading, impacting both investment and operational costs. As shown in Table 1, the MPR of 2.5 $\frac{L_{{CH}_{4}}}{L_{R}*d}$ achieved in this study compares well with studies of various reactors operated under similar conditions, although the rate we achieved is in the lower range. In previous research, MPRs ranging between 1.3 and 15.4 $\frac{L_{{CH}_{4}}}{L_{R}*d}$ have been reported. The highest MPR of 15.4 $\frac{L_{{CH}_{4}}}{L_{R}*d}$ was achieved by Strübing et al. after more than 305 days of operation. They reached an MPR of 1.3 $\frac{L_{{CH}_{4}}}{L_{R}*d}$ after 41 days and an MPR of 5.6 $\frac{L_{{CH}_{4}}}{L_{R}*d}$ after 73 days [40]. This demonstrates the significant impact of adaptation and growth duration on the MPR. The total duration of our study was 98 days, while the start-up phase was only 73 days, including an unstable phase before day 32.

The type of reactor system used in our study has been barely investigated before for its application in biological methanation. By contrast, hollow fiber membrane reactors, trickle-bed reactors, and conventional bubble column reactors have been well studied. Considering these factors, the results presented here can be considered promising for future biomethanation research.

### 3.2. Flexible Phase: Wind + PV Regime

**Figure 3:**
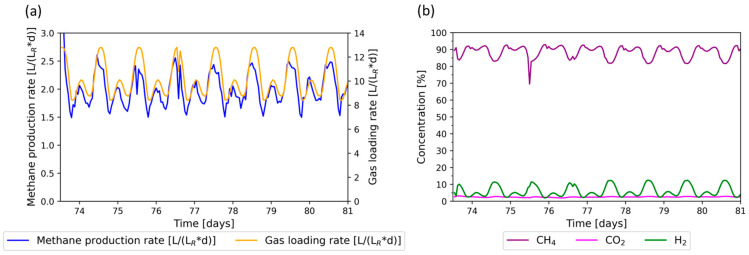
Methane production rate (MPR) during the flexible feed regime, based on a combined wind power + PV regime. (**a**) Gas loading rate (orange) and methane production rate (blue); (**b**) product gas concentration of methane (purple), carbon dioxide (magenta), and hydrogen (green).

As described in Section 2.5, the Wind + PV regime was tested first. Results in terms of the MPR (a) and gas composition (b) are shown in Figure 3. The lowest gas loading rate was 8.8 $\frac{L_{gas}}{L_{R}*d}$. and the highest was 12.8 $\frac{L_{gas}}{L_{R}*d}.$ The MPR follows the trend of the gas loading rate, with a minimum MPR of 1.5 $\frac{L_{{CH}_{4}}}{L_{R}*d}$ and a maximum of 2.5 $\frac{L_{{CH}_{4}}}{L_{R}*d}$. The lowest hydrogen concentration detected was 2.0%, and the highest was 12.5%. The carbon dioxide concentration ranged between 1.9% and 2.8%. Methane concentrations ranged between 81.7% and 92.8% but dropped to 69.5% between days 75 and 76. The fluctuation of the loading rate did not have a negative impact on methane production. The limitations in relation to the decreasing methane concentration and increasing hydrogen at high gas loading rates were identified during the initial phase and persisted.

### 3.3. Flexible Phase: PV Regime

**Figure 4:**
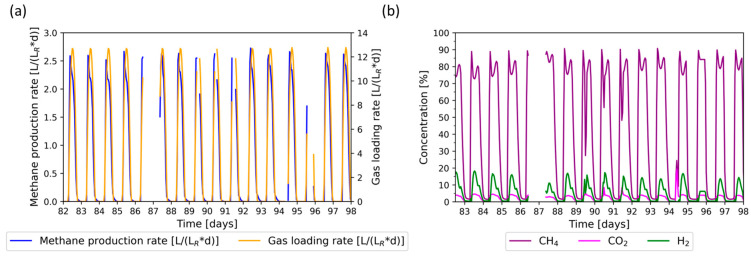
Methane production rate (MPR) during the flexible feed regime, based on a photovoltaic (PV) regime. (**a**) Gas loading rate (orange) and methane production rate (blue); (**b**) product gas concentration of methane (purple), carbon dioxide (magenta), and hydrogen (green).

After successfully testing the Wind + PV regime, the PV regime was implemented. The MPR (a) and gas composition (b) are shown in Figure 4. Due to technical issues, there were phases with interrupted data transmission. The gas loading rate varied from 0 $\frac{L_{gas}}{L_{R}*d}$ to 12.8 $\frac{L_{gas}}{L_{R}*d}$. The MPR followed the trend of the gas loading rate, with the highest MPR recorded as 2.7 $\frac{L_{{CH}_{4}}}{L_{R}*d}$. The highest methane concentrations were measured during the ramp-up phase of the loading rate (90.7%). Due to the high gas fraction in the reactor, gas remains in the system when the gas loading rate is 0 $\frac{L_{gas}}{L_{R}*d}$, and this is converted to methane. During the ramp-up of the gas loading rate, the high concentration of methane gas is expelled from the system, resulting in an initial methane peak for each ramp-up phase. At the peak gas loading rate, the methane concentration decreases to as little as 71.6%, subsequently increasing to as much as 87.2% as the gas loading rate reduces. Hydrogen and carbon dioxide concentrations increase with each increment of the gas loading rate, reaching up to 18.2% hydrogen and up to 4.6% carbon dioxide.

The data provided indicate that the new reactor design is capable of PV-powered methanation. Periods of downtime lasting 12 h resulted in minimal disturbance to the process. Although there was an increase in the hydrogen concentration at higher gas loading rates, the MPR maintained at a high level and did not deviate notably from the measurements taken during the initial phase and the Wind + PV regime. However, the concentration of hydrogen often exceeded the limit for direct injection into most gas grids without additional purification, as the limits usually range from 5% to 10% [48]. It is probable that reducing the gas loading rate would lower the hydrogen concentrations at the gas loading rate peak, ultimately decreasing the fluctuation of the methane concentration.

Trickle-bed reactor tests at 55 °C have demonstrated that the recovery time after downtimes of 12–24 h ranges from 1.5 to 7 h [17,18]. The recovery time in our study appears to be minimal. However, we observed fluctuating methane concentrations. A study by Aghtaei et al. demonstrated that, under similar feeding conditions, the methane concentration in a 0.5 L continuous-stirred tank reactor operated at 40 °C fluctuated from around 70% to 90% [19]. The new reactor design presented in our study is suitable for flexible biomethanation, demonstrating a fast recovery after downtime with an MPR comparable to other biomethanation designs operated under similar conditions.

### 3.4. Growth and Process Stability

**Figure 5:**
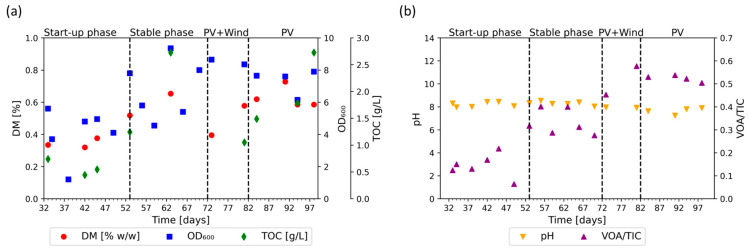
Growth and stability parameters during the initial phase, consisting of a start-up phase and stable phase, and the flexible phase, consisting of photovoltaic (PV) + Wind and PV-only regimes; (**a**) growth parameters: dry matter (DM [%], red dots), optical density (OD_600_, blue squares), and total organic carbon (TOC [g/L], green diamonds); (**b**) stability parameters: pH (orange triangles pointing downwards) and volatile organic acid to total inorganic carbonate ratio VOA/TIC (purple triangles pointing upwards).

The DM, TOC, and the OD_600_ were measured in order to monitor microbial growth. The results are shown in Figure 5a. During the initial phase, all three parameters increased. The DM rose to 0.65%, the TOC reached 2.7 g/L, and the OD_600_ increased to 9.35, showing high fluctuations. During the flexible phase, both the DM and TOC initially decreased by about 50%, but then recovered. The OD_600_ remained at the same level and slightly decreased towards the end of the flexible phase. This indicates exponential microbial growth during the initial phase and its adaptation at the beginning of the flexible phase. Although the introduction of a flexible feeding structure results in a static behavior of biomass growth, the concentration of biomass remained stable, and no degradation was observed.

There are limitations with regard to the analytical techniques we employed, however. Inorganic compounds can impact the DM and VOA, while other secondary metabolites can affect the TOC. Furthermore, cell density and size can influence the OD_600_ measurement [49,50]. Nonetheless, the increase in DM, TOC, and OD_600_ denotes microbial growth.

Furthermore, pH and the VOA/TIC were measured to investigate the stability and the buffer capacity of the system. The results are shown in Figure 5b. The pH remains stable, at an average of pH 8.1 ± 0.25, during the initial phase and the flexible phase, while the VOA/TIC increases. The VOA/TIC reached a plateau between days 53 and 72, with an average of 0.33 ± 0.05. It increased again during the flexible phase, reaching a plateau between days 81 and 98 with an average of 0.52 ± 0.04. This indicates an increased formation of volatile organic acids.

The VOA/TIC can be used as a parameter to observe the process stability of anaerobic digestion in terms of volatile organic acid formation and acidification [51]. Although the VOA/TIC values have increased, the pH value remained relatively stable, which indicates that the buffer capacity was still sufficient. It has been reported that even high concentrations of volatile organic acids do not have strong inhibitory effects on biogas processes, provided the buffer is sufficient [52]. In this respect, the high concentration of nitrogen in the form of urea and diammonium phosphate appears to be sufficient to provide the required buffer capacity in our system. Ammonia is a well-suited buffer for biogas and methanation processes [17,21].

During the initial 32 days, the process was unstable, with high VOA/TIC values reaching up to 3.1, resulting in pH values of up to 5.8 (see Supplementary Materials, Figure S4). The modified nutrient supplementation resulted in enhanced process stability. The process became stable after adjusting the supplementation strategy by adding more inoculum on days 13 and 28. Further automation of the system should include an automated dosing of supplements.

### 3.5. Microbial Community Structure

**Figure 6:**
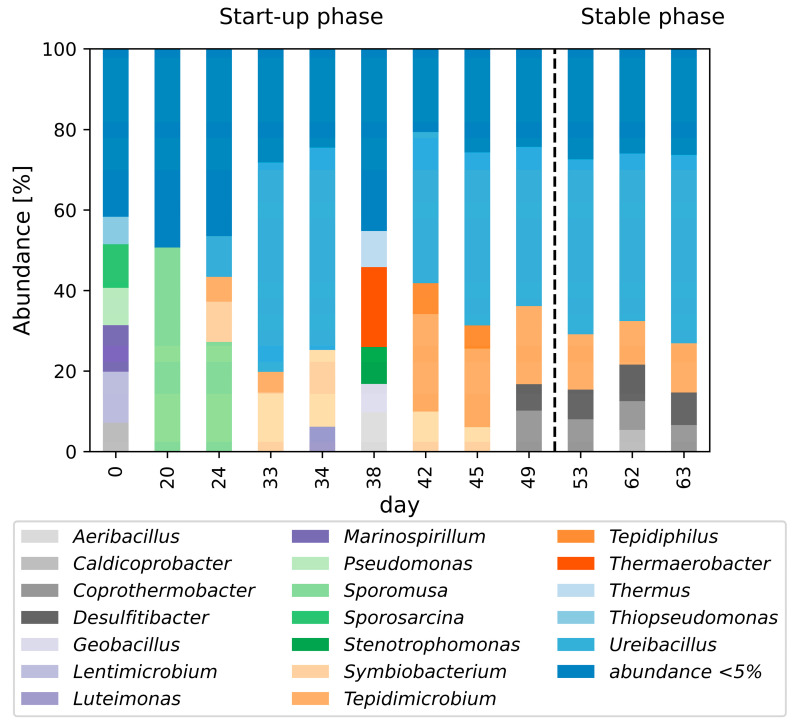
Bacterial community structure analysis based on 16S rRNA gene V3–V4 region amplicon sequencing at the genus level, archaea excluded. Taxa with an abundance higher than 5% are shown. The sample on day 0 represents the inoculum. On days 13 and 28 additional inoculum was added. Bacterial community structure analysis with abundances higher than 1% can be found in the Supplementary Materials, Figure S5.

The composition of the microbial community can affect the MPR [15,53]. Therefore, the bacterial and the methanogenic microbial structure were analyzed via amplicon sequencing data. The bacterial structure is shown in Figure 6.

The bacterial community structure in the inoculum was highly diverse (see Supplementary Materials, Figure S6). This diversity decreased during the initial phase. Between day 20 (50%) and day 24 (27%), the genus *Sporomusa* of the phylum Bacillota was most abundant. In this period, the process was not stable: on day 20 the pH dropped to 5.8 (VOA/TIC = 3.1) and on day 24 it increased to 6.5 (VOA/TIC = 2.2), which was induced by formation of volatile organic acids. This could have been partially caused by the increased abundance of homoacetogenic *Sporomusa* [54,55].

From day 33, there was a shift in the bacterial community, presumably due to the added inoculum and adapted nutrient supplementation on day 28. Between days 33 and 34 and between days 42 and 63, *Ureibacillus* of the phylum Bacillota was the predominant bacterial genus, with 38–52% abundance. Bacteria affiliated with *Ureibacillus* are described as being strictly aerobic microorganisms with urease activity [56]. *Ureibacillus* are an indicator of oxygen being present in the process. Perhaps a small leak on the suction side of the pump led to the oxygen introduction. As no oxygen was detected in the product gas, it seems to have been metabolized in the reactor. *Ureibacillus* has been found in an anaerobic process designed for VOA production [57]. On day 38, the bacterial structure was different compared to the other days of the start-up phase. This is associated with the particularly low OD of 1.3 and the decrease in methane concentration observed on this day.

The abundance of the genus *Symbiobacterium* increased to 19% by day 34 and then decreased to 4.3% by day 63. *Symbiobacterium* belongs to the phylum Bacillota. Species of this genus are facultatively anaerobic, growing on hydrogen and carbon dioxide, preferably in symbiosis with *Geobacillus*, and are capable of nitrate reduction [58,59]. The abundance of the genus *Geobacillus* was above 1% only on day 24 and day 38, so the growth of *Symbiobacterium* seems to be mainly dependent on a carbon dioxide atmosphere in symbiosis with other organisms.

The abundance of the genus *Desulfitibacter* increased from less than 1% in the inoculum to 9% on day 62. *Desulfitibacter* belongs to the phylum Bacillota. Only one species is currently described that respires sulfite, thiosulfate, and sulfur and grows on yeast extract, but not on hydrogen and oxygen, nor on various VOAs and sugars [60]. Presumably, *Desulfitibacter* acted proteolytically in the process.

From day 42 to 48 the abundance of the genus *Coprothermobacter* increased from 1.6 to 10.2%, before it decreased to 6.6% by day 63. *Coprothermobacter* are strictly anaerobic and proteolytic, poorly fermenting sugars to produce acetate and hydrogen and carbon dioxide [61]. An abundance (up to 55%) of *Coprothermobacter* has previously been reported in other ex situ methanation processes [62].

The genus *Tepidimicrobium* was found with increasing abundance until day 42, when it reached its maximum level of 24.2%, decreasing to 12.2% by day 63. Species of the genus *Tepidimicrobium* belong to the phylum Bacillota and produce hydrogen, carbon dioxide, acetate, butyrate, and ethanol from proteins and various carbohydrates. *Tepidimicrobium* have also been found in an increased amount in a biogas process, where they were associated with the acetotrophic pathway [63].

The abundance of the genus *Tepidiphilus* of the phylum Pseudomonadota increased until day 42, reaching its maximum of 7.6% before decreasing to less than 1% by day 63. Species of the genus *Tepidiphilus* are aerobic and thermophilic but can also grow anaerobically in the presence of nitrate. They potentially produce acetate, and it has been reported that they could possibly produce hydrogen due to hydrogenase activity [64,65].

In addition, about 9% to 18% of the bacterial community consisted of genera with a less than 1% abundance. In addition to the large abundance of *Ureibacillus*, which played an unknown role in the process, proteolytic bacteria capable of producing intermediates such as acetate, hydrogen, and carbon dioxide, which can be used for methanation by archaea, were especially abundant.

**Figure 7:**
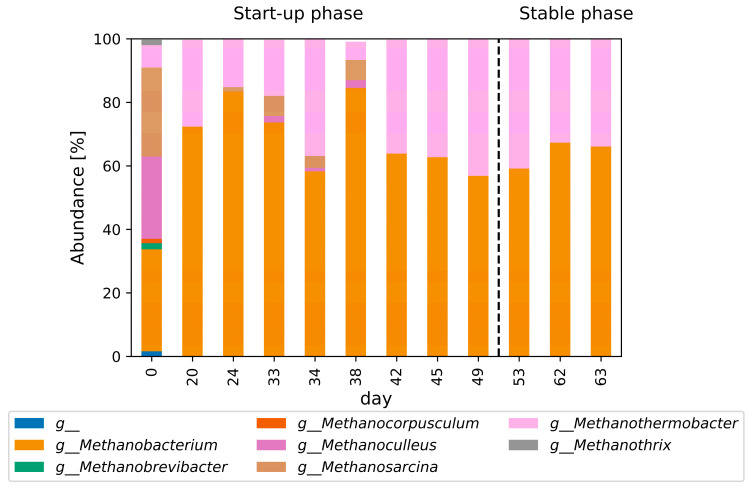
Methanogenic community composition on the genus level of the inoculum, the initial phase before inoculation and the initial phase after reinoculation. The sample on day 0 represents the inoculum, with g_(blue) representing the unknown genera detected.

The archaeal community structure on the genus level is shown in Figure 7. The inoculum was highly diverse (see Supplementary Materials, Figure S7), with an abundance of *Methanobacterium*, *Methanobrevibacter*, *Methanocorpusculum*. *Methanoculleus*, *Methanosarcina, Methanothermobacter*, and *Methanothrix.* The predominant genera were *Methanobacterium, Methanoculleus,* and *Methanosarcina.* In the start-up phase, the archaeal community structure shifted to a predominant abundance of the genera *Methanobacterium* and *Methanothermobacter.* During the start-up phase, low levels of *Methanosarcina* and *Methanoculleus* were also observed. In the stable phase, solely *Methanobacterium* and *Methanothermobacter* were detected by amplicon sequencing.

Members of the genus *Methanothermobacter* are described as strictly anaerobic, with optimal growth between 60 °C and 65 °C. They are mainly chemolithoautotrophic, reducing carbon dioxide with hydrogen to methane [66]. Ammonia serves as the sole nitrogen source for *Methanothermobacter* [67]. Species from the genus *Methanothermobacter*, such as *Methanothermobacter thermoautotrophicus* and *Methanothermobacter marburgensis*, show good growth yields and shorter doubling times (of 2 h) than other thermophilic archaea (doubling times from less than an hour to several days) [68,69]. They also perform outstandingly in terms of MPRs in continuous stirred-tank reactors compared with other methanogens [70]. *Methanothermobacter thermoautotrophicus* can be cultivated, resulting in an MPR of 40 $\frac{L_{{CH}_{4}}}{L_{R}*d}$ with a 59% methane content, while for *Methanothermobacter marburgensis* an MPR of 288 $\frac{L_{{CH}_{4}}}{L_{R}*d}$ with a methane content of 96% was reported [71,72].

Species of the genus *Methanobacterium* are strictly anaerobic and hydrogenotrophic, with optimum growth temperatures between 37 °C and 45 °C. Carbon dioxide is their predominant carbon source, while formate, secondary alcohols, and CO can be also used by some species [73]. Abundances of *Methanothermobacter* and *Methanobacterium* similar to those in our study have been reported for hydrogenotrophic mixed cultures grown at 55 °C [47,62,74]. In these studies, the species *Methanobacterium formicicum* of the genus *Methanobacterium* was found in greater abundances [47,74]. *Methanobacterium formicicum* accepts a variety of substrates for methanation, including acetate, carbohydrates, amino acids, ethanol, methanol, propionate, butyrate, and lactate [75].

## 4. Conclusions

Hydrogenotrophic biomethanation was successfully established in a new reactor system that had been described in an earlier study [20]. Considering the short adaptation period, the system shows promising results. An MPR of 2.5 $\frac{L_{{CH}_{4}}}{L_{R}*d}$ was achieved. During the initial and the flexible phase there were no signs of inhibiting acidification. The pH was very stable, at 8.1 ± 0.25. We showed that the new reactor design can compete with existing reactor designs under similar conditions. In terms of flexibility, it was shown that the reactor can be operated with downtimes of 12 h, with an immediate response after resuming the feeding. This allows the connection of biomethanation plants based on our design to PV or wind power plants. It is also possible to use the reactor for control measures for the power grid. Future tests should reveal its full potential by testing how fast the loading rate can be set to its maximum after a downtime. Further, the long-term stability of the process should be investigated, as with 73 days the operation was too short to see long-term effects.

In future, the reactor can be optimized further. The presence of the strictly aerobic *Ureibacillus* indicates that oxygen leaks into the system. Oxygen could be slightly inhibitory to the overall process, so improving oxygen tightness could thus improve performance. Nevertheless, the presence of strict anaerobes such as *Methanothermobacter* and *Methanobacterium* indicate appropriate conditions for methanogenesis. In addition, an optimized, fully automated nutrient feeding strategy with shorter feeding intervals could improve the microbial growth and the MPR. Improved static mixers could further improve mixing properties and thus reduce hydrogen mass transfer limitations, leading to an increased MPR. With a pressure-resistant design, pressure could also be increased, which would in turn increase the MPR. It would also be possible to use well-established pure cultures such as *Methanothermobacter thermoautotrophicus* to increase the MPR. In addition, by optimizing recirculation, parasitic energy consumption could be decreased.

Seeing the potential for further optimization and the flexibility of the system, the new design is a promising approach for sector coupling and as a grid-regulating measure.

## Figures and Tables

**Table 1:** The comparison of continuous biomethanation processes operated at 55 °C and inoculated with sludge from biogas plants or wastewater treatment plants (partially previously enriched hydrogenotrophic cultures were used).

Type	V_R_ [*L*]	*LR*_*H*_2__ [*L*_*H*_2__/ (*L* × *d*)]	CH_4_ [%]	MPR [*L*_*CH*_4__/(*L*_*R*_ × *d*)]	Feed Ratio H_2_:CH_4_:CO_2_	Recirculation (Liquid; Gas)	pH	Reference
HFM	31	40.2		**8.84**	4:0:1	no; 4.83 m^3^/d		[41]
HFM	60	30		**6.6**	4:0:1	60 L/h; 17.7 m^3^/d		[42]
TBR 2 Stage	2 ^1^/2 ^2^	**7.2**	90	1.73	N.a.	1 L/72 h; no	6.9 ^1^/6.7 ^2^	[43]
TBR	58.1	62.1	98	15.4	4:0:1	10 L/h; no	~7	[40]
TBR	1	**12.8**	95	**3.13**	4:0:1	yes; no	7–8	[44]
TBR	8.3	10.8	86	2.60	4:0:1	75 mL/min; no	8.5–9.3	[45]
TBR 2 Stage	1000 ^1^/1000 ^2^	41	90	10	62:22:16	0.7 m^3^/(m^3^R × h); no	8.5	[17]
TBR	58.1	52.5	97.5	13.1	3.78:0:1	3 L/h; no	7	[18]
BCR	9.5	73.3	15	9.1	4:0:1	No	7.4	[46]
BCR	1	**5.95**	87	1.3	62:23:15	no; 117 L/(*L*_*R*_ × day)	8.5	[47]
BCR	18	**14.5**	90	4	4.2:0:1	no; 120 L/(*L* × *d*)]	5.5–8	[16]
PFR	22.5	10.2	81–94	2.5	4:0:1	258 L/h; no	8.1	this study

V_R_ = Volume reactor; LR_H2_ = hydrogen loading rate; MPR = methane production rate; HFM = hollow fiber membrane; TBR = trickle-bed reactor; BCR = bubble column reactor; PFR = plug flow reactor. Bold results were calculated based on given data; Stage 1 ^1^, Stage 2 ^2^.

## Data Availability

The data are contained within the article and Supplementary Materials.

## Supplementary Materials

The following supporting information can be downloaded at https://www.mdpi.com/article/10.3390/bioengineering11020165/s1, Text S1: medium preparation, Text S2: compensation curve gas flow meter, Table S1: minimal medium composition, Table S2: trace elements SL10 (DSMZ medium 320), Table S3: trace elements SL10 (DSMZ medium 320), Table S4: Wind and PV regime, Table S5: PV regime, Figure S1: compensation curve gas flow meter, Figure S2: gas loading rate and product gas composition during the initial phase before day 32, Figure S3: N-total and P-total during the initial phase, consisting of the start-up phase and stable phase, and the flexible phase, consisting of the PV + Wind and PV regimes, Figure S4: stability parameters during the initial phase before day 32, Figure S5: bacterial community structure analysis based on 16S rRNA gene V3–V4 region amplicon sequencing at the genus level, archaea excluded, Figure S6: Shannon and Simpson indexes of the bacterial community structure, Figure S7: Shannon and Simpson indexes of the archaeal community structure.
