# Supplementary material for: Optimization of the Ex Situ Biomethanation of Hydrogen and Carbon Dioxide in a Novel Meandering Plug Flow Reactor: Start-Up Phase and Flexible Operation
Source: Bioengineering (Basel). 2024 Feb 8;11(2):165. doi: 10.3390/bioengineering11020165 (PMC10886298; doi:10.3390/bioengineering11020165)
Supplement: Supplementary file 1 [file bioengineering-11-00165-s001.zip › bioengineering-2862615-supplementary.pdf]

bioengineering

# Supplementary elements

**Text S1.** Medium preparation

**Text S2.** Compensation curve gas flow meter

**Table S1.** Minimal medium composition

**Table S2.** Trace elements SL10 (DSMZ medium 320)

**Table S3.** Trace elements SL10 (DSMZ medium 320)

**Table S4.** Wind and PV regime

**Table S5.** PV regime

**Figure S1.** Compensation curve gas flow meter

**Figure S2.** Gas loading rate and product gas composition during the initial phase before day 32

**Figure S3.** N-total and P-total during the initial phase consisting of start-up phase and stable phase and the flexible phase consisting of PV+Wind and PV regime

**Figure S4.** Stability parameters during the initial phase before day 32

**Figure S5.** Bacterial community structure analysis based on 16S rRNA gene V3-V4 region amplicon sequencing on genus level, Archaea excluded.

**Figure S6.** Shannon and Simpson indexes of the bacterial community structure.

**Figure S7.** Shannon and Simpson indexes of the archaeal community structure.

**Text S1.** Medium preparation

Minimal medium for the cultivation of a mixed culture was prepared according to Table 1. Diammoniumphosphate and Urea were prepared freshly. The pH was controlled after media preparation and was 8.0 ±0.1.

The trace element stock solution Trace elements SL10 (DSMZ medium 320) was prepared first by dissolving $FeCl_2$ in HCl and diluting it with water before other salts were added and dissolved according to Table S2. The Selenite-tungstate stock solution (DSMZ medium 385) was prepared according to Table S3. Both stock solutions were stored in sterile serum bottles after sterile filtration with 0.2 µm PES filter (VWR International GmbH, Darmstadt, Germany) at 7°C.

A sodium sulfide stock solution (c= 50 g/L) was prepared by dissolving sodium sulfide nonahydrate in water and adjusting the pH to 7.0 with NaOH and HCL. The sodium sulfide stock solution was aliquoted in 50 ml tubes and stored at -20°C.

**Table S1.** Minimal medium composition

| Component | Concentra-tion | Unit |
| --- | --- | --- |
| Urea | 1.7560 | g/L |
| Diammoniumphosphate | 0.8527 | g/L |
| Sodium sulfate stock solution | 7.50 | ml/L |
| Trace element stock solution SL-10 (DSMZ medium 320) | 1.00 | ml/L |
| Selenite-tungstate stock solution (DSMZ medium 385) | 1.00 | ml/L |

**Table S2**. Trace elements SL10 (DSMZ medium 320)

| component | Concentra-tion | unit |
| --- | --- | --- |
| HCl (25%) | 10 | ml |
| FeCl2 x 4 H2O | 1500 | mg/L |
| ZnCl2 | 70 | mg/L |
| MnCl2 x 4 H2O | 100 | mg/L |
| H3BO3 | 6 | mg/L |
| CoCl2 x 6 H2O | 190 | mg/L |
| CuCl2 x 2 H2O | 2 | mg/L |
| NiCl2 x 6 H2O | 24 | mg/L |
| Na2MoO4 x 2 H2O | 36 | mg/L |

**Table S3.** Trace elements SL10 (DSMZ medium 320)

| Component | Concentra-tion | unit |
| --- | --- | --- |
| NaOH | 500 | mg/L |
| Na2SeO3 x 5 H2O | 3 | mg/L |
| Na2WO4 x 2 H2O | 4 | mg/L |

**Table S4.** Wind and PV regime

| start_time | end_time | setpoint |
| --- | --- | --- |
| 00:00:00 | 00:14:59 | 0 |
| 00:15:00 | 00:29:59 | 0 |
| 00:30:00 | 00:44:59 | 0 |
| 00:45:00 | 00:59:59 | 0 |
| 01:00:00 | 01:14:59 | 0 |
| 01:15:00 | 01:29:59 | 0 |
| 01:30:00 | 01:44:59 | 0 |

| | | |
|---|---|---|
| 01:45:00 | 01:59:59 | 0 |
| 02:00:00 | 02:14:59 | 0 |
| 02:15:00 | 02:29:59 | 0 |
| 02:30:00 | 02:44:59 | 0 |
| 02:45:00 | 02:59:59 | 0 |
| 03:00:00 | 03:14:59 | 0 |
| 03:15:00 | 03:29:59 | 0 |
| 03:30:00 | 03:44:59 | 0 |
| 03:45:00 | 03:59:59 | 0 |
| 04:00:00 | 04:14:59 | 0 |
| 04:15:00 | 04:29:59 | 0 |
| 04:30:00 | 04:44:59 | 0 |
| 04:45:00 | 04:59:59 | 0 |
| 05:00:00 | 05:14:59 | 0 |
| 05:15:00 | 05:29:59 | 0 |
| 05:30:00 | 05:44:59 | 0 |
| 05:45:00 | 05:59:59 | 0 |
| 06:00:00 | 06:14:59 | 0 |
| 06:15:00 | 06:29:59 | 0 |
| 06:30:00 | 06:44:59 | 0 |
| 06:45:00 | 06:59:59 | 0 |
| 07:00:00 | 07:14:59 | 48 |
| 07:15:00 | 07:29:59 | 77 |
| 07:30:00 | 07:44:59 | 126 |
| 07:45:00 | 07:59:59 | 185 |
| 08:00:00 | 08:14:59 | 248 |
| 08:15:00 | 08:29:59 | 314 |
| 08:30:00 | 08:44:59 | 383 |
| 08:45:00 | 08:59:59 | 455 |
| 09:00:00 | 09:14:59 | 524 |
| 09:15:00 | 09:29:59 | 593 |
| 09:30:00 | 09:44:59 | 654 |
| 09:45:00 | 09:59:59 | 707 |
| 10:00:00 | 10:14:59 | 753 |
| 10:15:00 | 10:29:59 | 793 |
| 10:30:00 | 10:44:59 | 825 |
| 10:45:00 | 10:59:59 | 853 |
| 11:00:00 | 11:14:59 | 877 |
| 11:15:00 | 11:29:59 | 895 |
| 11:30:00 | 11:44:59 | 913 |
| 11:45:00 | 11:59:59 | 921 |
| 12:00:00 | 12:14:59 | 935 |
| 12:15:00 | 12:29:59 | 949 |
| 12:30:00 | 12:44:59 | 957 |
| 12:45:00 | 12:59:59 | 959 |

| | | |
|---|---|---|
| 13:00:00 | 13:14:59 | 960 |
| 13:15:00 | 13:29:59 | 955 |
| 13:30:00 | 13:44:59 | 944 |
| 13:45:00 | 13:59:59 | 932 |
| 14:00:00 | 14:14:59 | 914 |
| 14:15:00 | 14:29:59 | 898 |
| 14:30:00 | 14:44:59 | 884 |
| 14:45:00 | 14:59:59 | 860 |
| 15:00:00 | 15:14:59 | 829 |
| 15:15:00 | 15:29:59 | 793 |
| 15:30:00 | 15:44:59 | 761 |
| 15:45:00 | 15:59:59 | 715 |
| 16:00:00 | 16:14:59 | 670 |
| 16:15:00 | 16:29:59 | 617 |
| 16:30:00 | 16:44:59 | 561 |
| 16:45:00 | 16:59:59 | 503 |
| 17:00:00 | 17:14:59 | 444 |
| 17:15:00 | 17:29:59 | 384 |
| 17:30:00 | 17:44:59 | 322 |
| 17:45:00 | 17:59:59 | 261 |
| 18:00:00 | 18:14:59 | 201 |
| 18:15:00 | 18:29:59 | 146 |
| 18:30:00 | 18:44:59 | 99 |
| 18:45:00 | 18:59:59 | 61 |
| 19:00:00 | 19:14:59 | 48 |
| 19:15:00 | 19:29:59 | 0 |
| 19:30:00 | 19:44:59 | 0 |
| 19:45:00 | 19:59:59 | 0 |
| 20:00:00 | 20:14:59 | 0 |
| 20:15:00 | 20:29:59 | 0 |
| 20:30:00 | 20:44:59 | 0 |
| 20:45:00 | 20:59:59 | 0 |
| 21:00:00 | 21:14:59 | 0 |
| 21:15:00 | 21:29:59 | 0 |
| 21:30:00 | 21:44:59 | 0 |
| 21:45:00 | 21:59:59 | 0 |
| 22:00:00 | 22:14:59 | 0 |
| 22:15:00 | 22:29:59 | 0 |
| 22:30:00 | 22:44:59 | 0 |
| 22:45:00 | 22:59:59 | 0 |
| 23:00:00 | 23:14:59 | 0 |
| 23:15:00 | 23:29:59 | 0 |
| 23:30:00 | 23:44:59 | 0 |
| 23:45:00 | 23:59:59 | 0 |

**Table S5.** PV regime

| start_time | end_time | setpoint |
|---|---|---|
| 00:00:00 | 00:14:59 | 0 |
| 00:15:00 | 00:29:59 | 0 |
| 00:30:00 | 00:44:59 | 0 |
| 00:45:00 | 00:59:59 | 0 |
| 01:00:00 | 01:14:59 | 0 |
| 01:15:00 | 01:29:59 | 0 |
| 01:30:00 | 01:44:59 | 0 |
| 01:45:00 | 01:59:59 | 0 |
| 02:00:00 | 02:14:59 | 0 |
| 02:15:00 | 02:29:59 | 0 |
| 02:30:00 | 02:44:59 | 0 |
| 02:45:00 | 02:59:59 | 0 |
| 03:00:00 | 03:14:59 | 0 |
| 03:15:00 | 03:29:59 | 0 |
| 03:30:00 | 03:44:59 | 0 |
| 03:45:00 | 03:59:59 | 0 |
| 04:00:00 | 04:14:59 | 0 |
| 04:15:00 | 04:29:59 | 0 |
| 04:30:00 | 04:44:59 | 0 |
| 04:45:00 | 04:59:59 | 0 |
| 05:00:00 | 05:14:59 | 0 |
| 05:15:00 | 05:29:59 | 0 |
| 05:30:00 | 05:44:59 | 0 |
| 05:45:00 | 05:59:59 | 0 |
| 06:00:00 | 06:14:59 | 0 |
| 06:15:00 | 06:29:59 | 0 |
| 06:30:00 | 06:44:59 | 0 |
| 06:45:00 | 06:59:59 | 0 |
| 07:00:00 | 07:14:59 | 0 |
| 07:15:00 | 07:29:59 | 0 |
| 07:30:00 | 07:44:59 | 0 |
| 07:45:00 | 07:59:59 | 0 |
| 08:00:00 | 08:14:59 | 0 |
| 08:15:00 | 08:29:59 | 0 |
| 08:30:00 | 08:44:59 | 0 |
| 08:45:00 | 08:59:59 | 0 |
| 09:00:00 | 09:14:59 | 0 |
| 09:15:00 | 09:29:59 | 0 |
| 09:30:00 | 09:44:59 | 0 |
| 09:45:00 | 09:59:59 | 0 |
| 10:00:00 | 10:14:59 | 0 |
| 10:15:00 | 10:29:59 | 0 |
| 10:30:00 | 10:44:59 | 0 |

| | | |
|---|---|---|
| 10:45:00 | 10:59:59 | 0 |
| 11:00:00 | 11:14:59 | 0 |
| 11:15:00 | 11:29:59 | 0 |
| 11:30:00 | 11:44:59 | 0 |
| 11:45:00 | 11:59:59 | 0 |
| 12:00:00 | 12:14:59 | 77 |
| 12:15:00 | 12:29:59 | 126 |
| 12:30:00 | 12:44:59 | 185 |
| 12:45:00 | 12:59:59 | 248 |
| 13:00:00 | 13:14:59 | 314 |
| 13:15:00 | 13:29:59 | 383 |
| 13:30:00 | 13:44:59 | 455 |
| 13:45:00 | 13:59:59 | 524 |
| 14:00:00 | 14:14:59 | 593 |
| 14:15:00 | 14:29:59 | 654 |
| 14:30:00 | 14:44:59 | 707 |
| 14:45:00 | 14:59:59 | 753 |
| 15:00:00 | 15:14:59 | 793 |
| 15:15:00 | 15:29:59 | 825 |
| 15:30:00 | 15:44:59 | 853 |
| 15:45:00 | 15:59:59 | 877 |
| 16:00:00 | 16:14:59 | 895 |
| 16:15:00 | 16:29:59 | 913 |
| 16:30:00 | 16:44:59 | 921 |
| 16:45:00 | 16:59:59 | 935 |
| 17:00:00 | 17:14:59 | 949 |
| 17:15:00 | 17:29:59 | 957 |
| 17:30:00 | 17:44:59 | 959 |
| 17:45:00 | 17:59:59 | 960 |
| 18:00:00 | 18:14:59 | 955 |
| 18:15:00 | 18:29:59 | 944 |
| 18:30:00 | 18:44:59 | 932 |
| 18:45:00 | 18:59:59 | 914 |
| 19:00:00 | 19:14:59 | 898 |
| 19:15:00 | 19:29:59 | 884 |
| 19:30:00 | 19:44:59 | 860 |
| 19:45:00 | 19:59:59 | 829 |
| 20:00:00 | 20:14:59 | 793 |
| 20:15:00 | 20:29:59 | 761 |
| 20:30:00 | 20:44:59 | 715 |
| 20:45:00 | 20:59:59 | 670 |
| 21:00:00 | 21:14:59 | 617 |
| 21:15:00 | 21:29:59 | 561 |
| 21:30:00 | 21:44:59 | 503 |
| 21:45:00 | 21:59:59 | 444 |

| | | |
|---|---|---|
| 22:00:00 | 22:14:59 | 384 |
| 22:15:00 | 22:29:59 | 322 |
| 22:30:00 | 22:44:59 | 261 |
| 22:45:00 | 22:59:59 | 201 |
| 23:00:00 | 23:14:59 | 146 |
| 23:15:00 | 23:29:59 | 99 |
| 23:30:00 | 23:44:59 | 61 |
| 23:45:00 | 23:59:59 | 0 |

**Text 2**. Compensation curve gas flow meter

Correction factors were created in 10% increments from 0% to 100% Methane and plotted against the methane concentration. It was assumed that carbon dioxide and hydrogen react stoichiometrically to produce methane, so that the remaining gas contains hydrogen and carbon dioxide in a 4:1 ratio. The equation of the linear regression was then used to correct the gas volume flow based on the methane concentration.

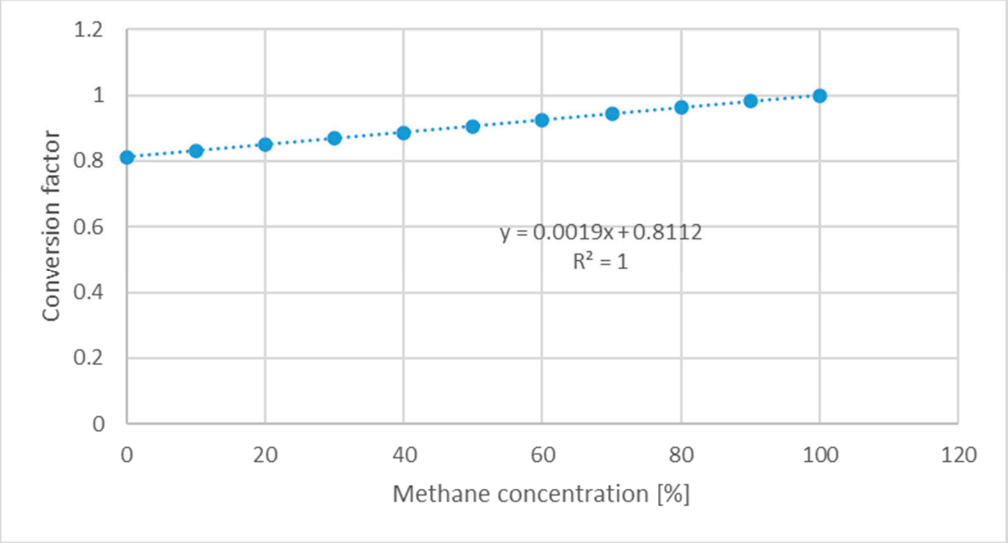


**Figure S1.** Compensation curve gas flow meter

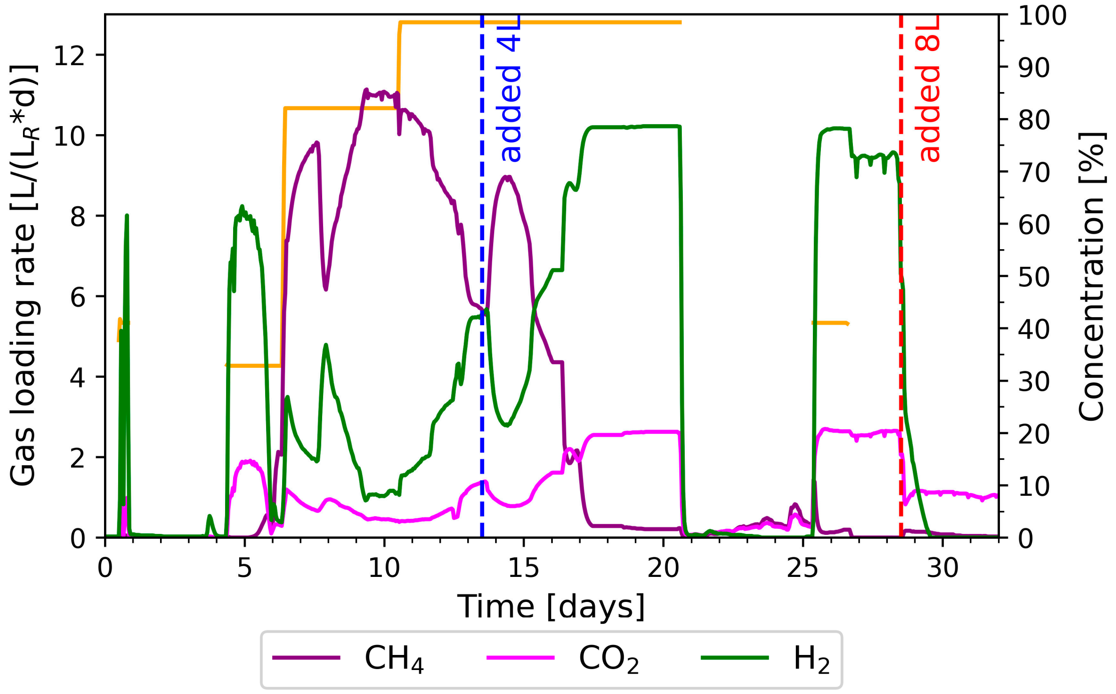


**Figure S2.** Gas loading rate (orange) and product gas concentration of methane (purple), carbon dioxide (magenta), hydrogen (green) during the initial phase before day 32.

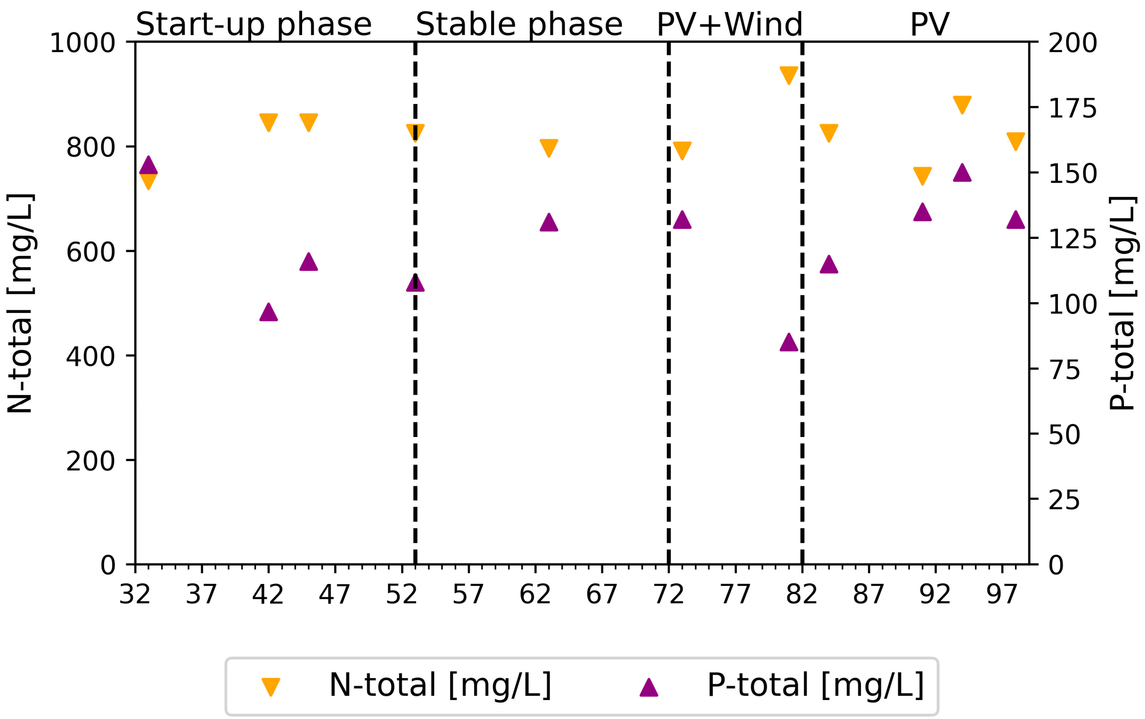


**Figure S3.** N-total (orange) and P-total (purple) during the initial phase consisting of start-up phase and stable phase (Max. LR) and the flexible phase consisting of PV+Wind and PV regime

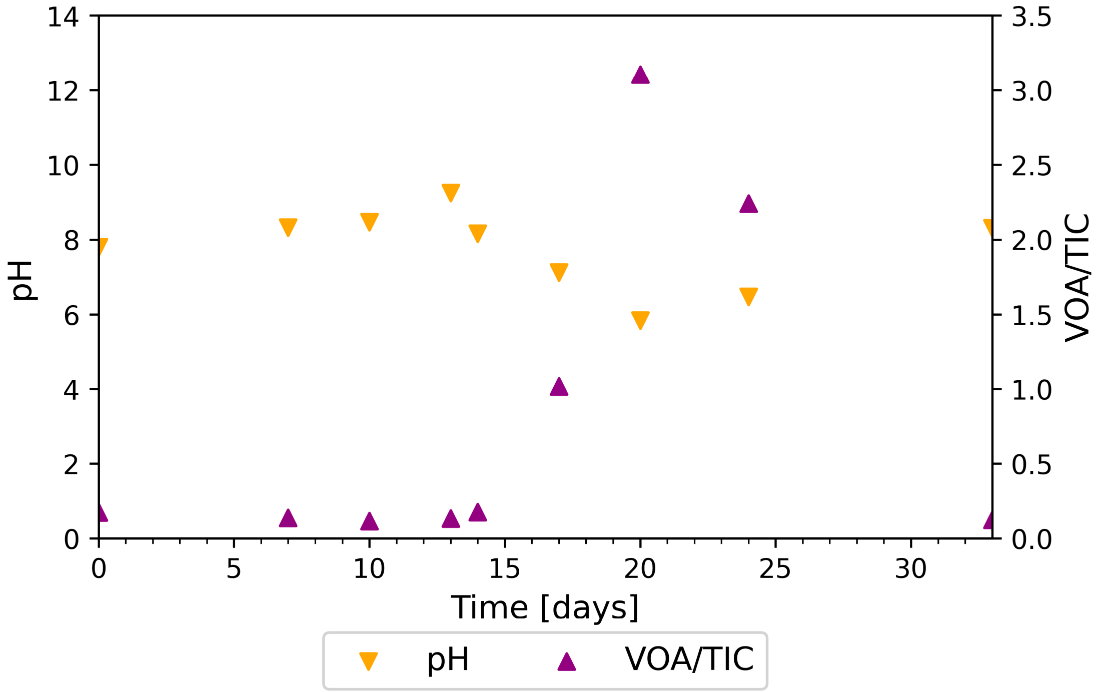


**Figure S4** Stability parameters during the initial phase before day 32 pH (orange triangles, tips pointing downwards) volatile organic acid to total inorganic carbonate ratio VOA/TIC (purple triangles, tips pointing upwards)

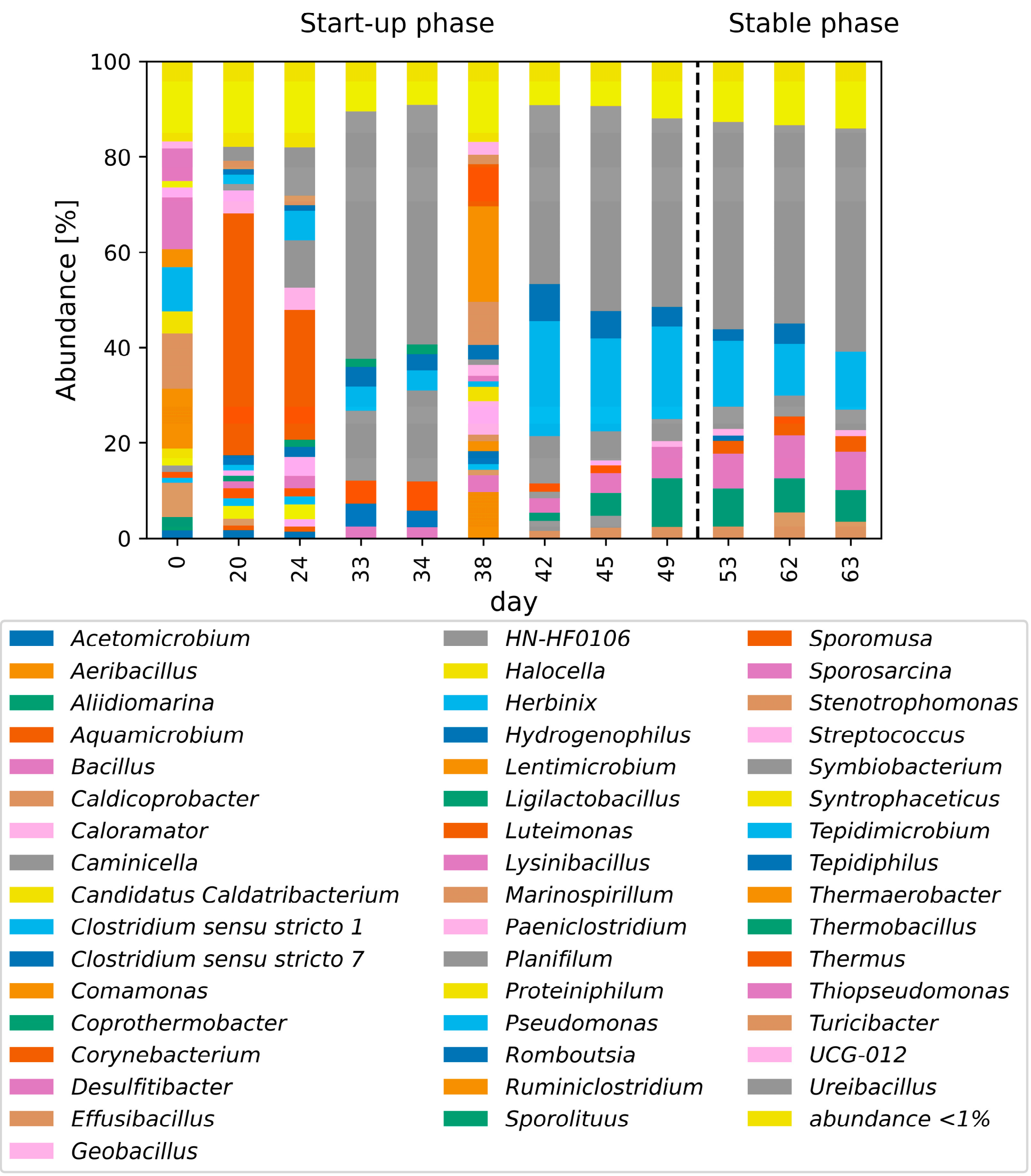


**Figure S5.** Bacterial community structure analysis based on 16S rRNA gene V3-V4 region amplicon sequencing on genus level, Archaea excluded. Taxa with abundance higher than 1%. The sample on day 0 represents the inoculum. On day 13 and 28 additional inoculum was added.

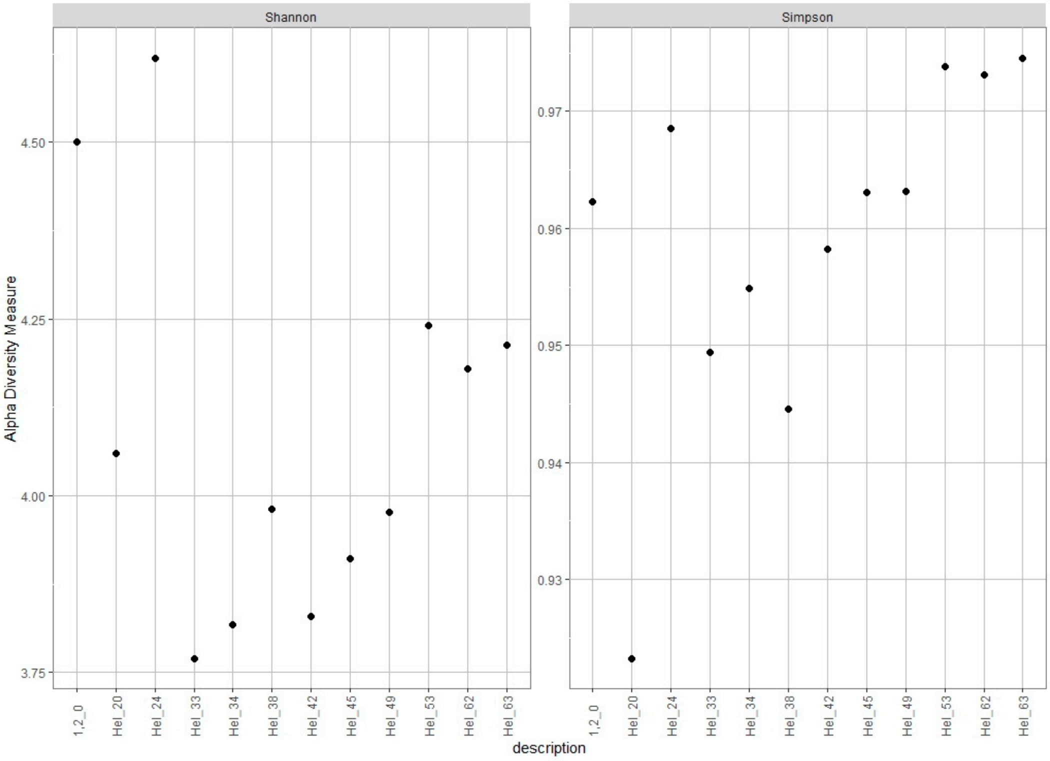


**Figure S6.** Shannon and Simpson indexes of the bacterial community structure. The number behind the suffix Hel_ and 1,2_ represents the day of operation. The sample on day 0 represents the inoculum.

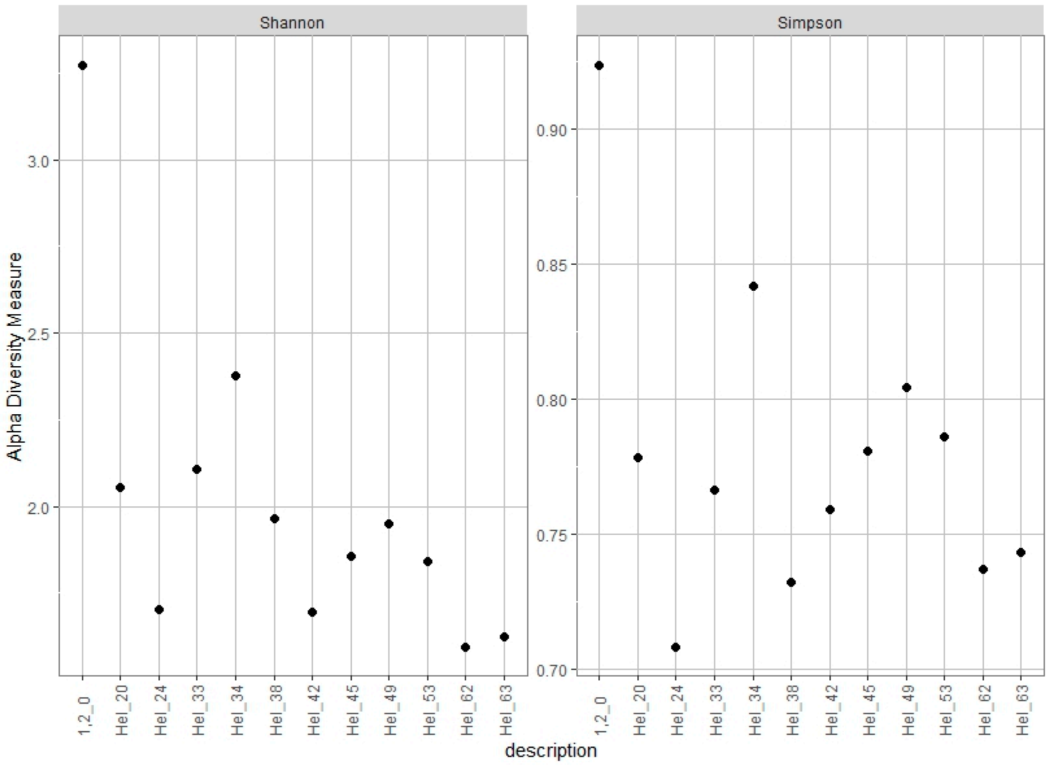


**Figure S7.** Shannon and Simpson indexes of the archaeal community structure. The number behind the suffix Hel_ and 1,2_ represents the day of operation. The sample on day 0 represents the inoculum.
